# Sex Differences in Flexibility-Arterial Stiffness Relationship and Its Application for Diagnosis of Arterial Stiffening: A Cross-Sectional Observational Study

**DOI:** 10.1371/journal.pone.0113646

**Published:** 2014-11-26

**Authors:** Masato Nishiwaki, Kazumichi Kurobe, Atsushi Kiuchi, Tomohiro Nakamura, Naoyuki Matsumoto

**Affiliations:** 1 Faculty of Engineering, Osaka Institute of Technology, Osaka, Japan; 2 Faculty of Business, Sports Management Course, Hannan University, Osaka, Japan; 3 Faculty of Health and Sport Sciences, University of Tsukuba, Tsukuba, Ibaraki, Japan; 4 Faculty of Environmental Symbiotic Sciences, Prefectural University of Kumamoto, Kumamoto, Japan; Morehouse School of Medicine, United States of America

## Abstract

**Purpose:**

Arterial stiffness might be related to trunk flexibility in middle-aged and older participants, but it is also affected by age, sex, and blood pressure. This cross-sectional observational study investigated whether trunk flexibility is related to arterial stiffness after considering the major confounding factors of age, sex, and blood pressure. We further investigated whether a simple diagnostic test of flexibility could be helpful to screen for increased arterial stiffening.

**Methods:**

According to age and sex, we assigned 1150 adults (male, n = 536; female, n = 614; age, 18–89 y) to groups with either high- or poor-flexibility based on the sit-and-reach test. Arterial stiffness was assessed by cardio-ankle vascular index.

**Results:**

In all categories of men and in older women, arterial stiffness was higher in poor-flexibility than in high-flexibility (P<0.05). This difference remained significant after normalizing arterial stiffness for confounding factors such as blood pressure, but it was not found among young and middle-aged women. Stepwise multiple-regression analysis also supported the notion of the sex differences in flexibility-arterial stiffness relationship. Receiver operating characteristic curve analysis revealed that cut-off values for sit-and-reach among men and women were 33.2 (area under the curve [AUC], 0.711; 95% confidence interval [CI], 0.666–0.756; sensitivity, 61.7%; specificity, 69.7%) and 39.2 (AUC, 0.639; 95% CI, 0.592–0.686; sensitivity, 61.1%; specificity, 62.0%) cm, respectively.

**Conclusion:**

Our results indicate that flexibility-arterial stiffness relationship is not affected by BP, which is a major confounding factor. In addition, sex differences are observed in this relationship; poor trunk flexibility increases arterial stiffness in young, middle-aged, and older men, whereas the relationship in women is found only in the elderly. Also, the sit-and-reach test can offer a simple method of predicting arterial stiffness at home or elsewhere.

## Introduction

Arterial stiffness is identified as an independent risk factor for future cardiovascular disease [1]. Previous reports indicate that arterial stiffness increases with age and that arterial stiffening impairs the ability of arteries to buffer the pulsation of blood pressure and flow [2], [3]. Therefore, the prevention of arterial stiffness is of paramount importance.

Recent studies indicate that a stretching program might reduce arterial stiffness [4] and that poor trunk flexibility might be associated with greater arterial stiffening in middle-aged and older people [5]. However, two major issues require resolution. First, blood pressure (BP) strongly affects pulse wave velocity (PWV) as an index of arterial stiffness [6]. A recent meta-analysis has recommended yoga as an intervention for reducing BP [7], suggesting that a regular stretching exercise *per se* might reduce BP and that trunk flexibility might be directly associated with BP. Since Yamamoto et al. compared two flexibility groups with different BP [5], their findings raise the question of whether trunk flexibility is actually related to arterial stiffness or rather, to BP. Secondly, sex strongly affects arterial stiffness. In general, PWV is significantly lower in premenopausal women than age-matched men, whereas the difference disappears upon reaching menopause; sex differences in age-related arterial stiffening are found [8], [9]. The previous study normalized arterial stiffness for sex using ANCOVA [5]. However, this method is still interpreted as being incomplete and remains controversial about relationship between flexibility and arterial stiffness because two-thirds of the participants in the study were women and sex is a nominal variable [5]. In addition, if flexibility is actually a predictor of arterial stiffening, trunk flexibility could be applied as a simple diagnostic test to screen for arterial stiffening. However, the diagnostic accuracy and optimal cut-off values for the sit-and-reach test remain unknown. Therefore, further detailed studies are required, after considering the influences of BP and sex, to analyze the relationship between trunk flexibility and arterial stiffness and to assess the applicability of trunk flexibility as a diagnostic test. However, these issues have not been addressed as far as we can ascertain.

Based on this information, we hypothesized that trunk flexibility is related to arterial stiffness after considering the influences of BP, but this relationship would differ according to age and sex. We further hypothesized that the relationship could be applied as a simple diagnostic test to screen for arterial stiffening. The present study tests these hypotheses.

## Methods

### Ethics statement

The purpose, procedures, and risks of the study were explained to each participant. All of them provided written informed consent before participating in the study, which was reviewed and approved by the Human Ethics Committee at the Osaka Institute of Technology (approval number; 2012-8) and proceeded in accordance with the guidelines of the Declaration of Helsinki.

### Participants

**Figure 1:**
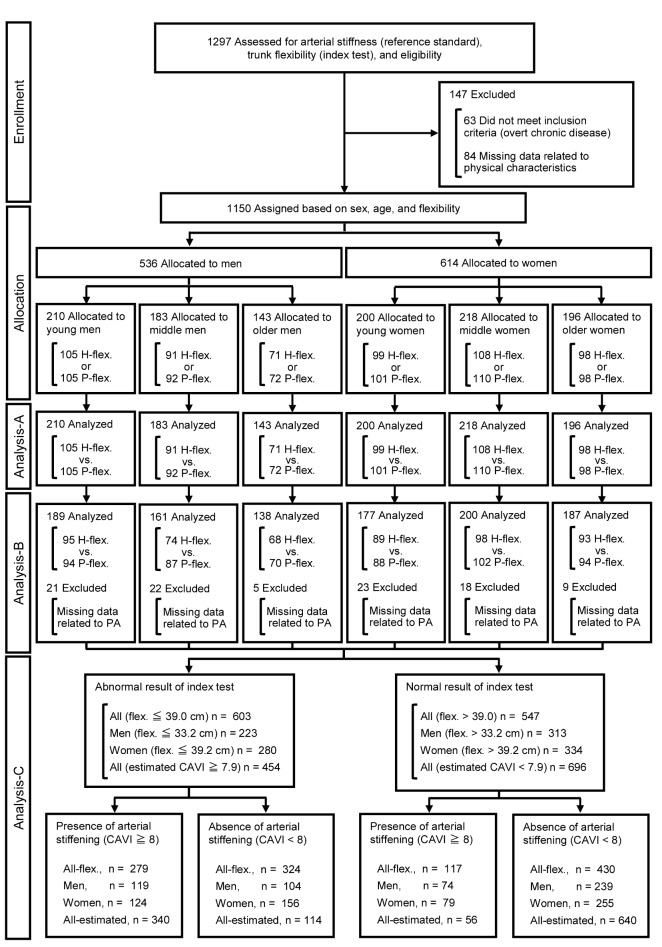
Flow diagram of study participants. Analysis-A comprised ANOVA, ANCOVA, and Pearson's correlation. Analysis-B comprised univariate and stepwise multiple regression analyses. Analysis-C comprised determination of diagnostic criteria using ROC curve analysis and then assessment of flexibility as a diagnostic test. CAVI, cardio-ankle vascular index of arterial stiffness; estimated CAVI, value of CAVI estimated by multiple regression equation; flex, flexibility; H-flex, high-flexibility; P-flex, poor-flexibility; PA, amount of physical activity.

We carried out this cross-sectional observational study of 1,297 Japanese volunteers (male, n = 589; female, n = 708; age, 18–89 y) in Osaka, Kumamoto, and Tokyo who participated in surveys of trunk flexibility and arterial stiffness between 2012 and 2013 (Figure 1). Participants were recruited from local advertisements, referrals, and from among the students and staff at the Osaka Institute of Technology, Setsunan University, and Prefectural University of Kumamoto. We excluded 63 participants with overt chronic diseases (BMI>30 kg/m^2^, cardiovascular diseases, diabetes mellitus, renal failure, and cancer) determined from medical histories and 84 with missing data related to arterial stiffness, flexibility, and handgrip strength (refusal to cooperate, back or hand pain, technical errors associated with measuring arterial stiffness such as not detecting the pulse wave of the first and second heart sounds, and one with an unmatched ID number). Thus, data were analyzed from 1,150 (male, n = 536; female, n = 614) healthy participants. None of the women were taking oral contraceptives or hormone replacement therapy [10].

### Sample size

We determined the appropriate sample size of each group before starting the study by power calculations using SPSS Sample Power (IBM, Tokyo, Japan). To assess the relationship between flexibility and arterial stiffness, participants in each age and sex category were categorized into groups with high- or poor-flexibility based on the median value of the sit-and-reach test. In accordance with previous findings [5], we assumed that the mean difference of arterial stiffness between the two groups would be 7.5% (approximately distributed between 5% and 10%). To detect this difference with 80% power and with a two-tailed α of 5%, each group should comprise 78 (>63) participants. To allow for possible correspondence with exclusion criteria and for comparisons with previous results, we planned to recruit about 100 participants per group (100 participants×2 groups×6 categories of age and sex = 1200). Thus, the present study included data from 1297 surveyed participants.

### Measurements

The participants abstained from vigorous exercise for at least 24 h to avoid the immediate effects of exercise on arterial stiffness, from smoking and medications on the day that the measurements were performed, and from caffeine and food for ≥4 h before testing [11], [12]. Firstly, we measured the height of the participants without footwear and weight in light clothing, and then calculated their body mass index (BMI), as weight divided by height squared. We also interviewed the participants to determine smoking status (no, 1; yes, 2), anti-hypertensive or anti-hyperlipidemic medications (yes, 1; no, 2). All values were measured in a quiet air-conditioned room at 22–24°C.

### Arterial stiffness as a reference standard, blood pressure, and heart rate

After resting for ≥15 min, the cardio-ankle vascular index (CAVI) as an index of arterial stiffness, BP, and heart rate (HR) were assessed using an automated device (VS-1500AE/AN, Fukuda Denshi, Tokyo, Japan) as described [13]–[18]. Electrocardiography (ECG), heart sounds, PWV, and BP were assessed in the supine position. Electrodes for ECG were placed on both wrists, and a microphone was placed at the sternum for phonocardiography. HR was automatically calculated from the R-R intervals on ECG. Cuffs were wrapped around both brachial upper arms and ankles and connected to a volume-plethysmographic sensor that determines volume pulse form and an oscillometric pressure sensor that measures BP. The automated oscillometric device recorded both the brachial BPs of supine participants and the procedure conformed strictly to American Heart Association guidelines [19]. As previously reported [15], [17], [18], [20], the CAVI values at the right and left sides were also automatically calculated using the following formula: CAVI = a [(2ρ/PP)×ln (SBP/DBP)×PWV^2^]+b, where SBP is systolic blood pressure, DBP is diastolic blood pressure, PP (pulse pressure) is SBP - DBP, ρ is the blood density, and a and b are constants. The means of the left and right brachial BP and CAVI values in each participant were subsequently analyzed. The CAVI represents arterial stiffness from the aorta to the ankle and is theoretically adjusted by BP [20]. Higher CAVI values mean stiffer arteries and the CAVI is therefore associated with risk for cardiovascular diseases with excellent validity and reproducibility [15], [17], [18], [21]. The day-to-day coefficient of variation (CV) determined in 10 individuals at our laboratory on two separate days was 3.6±0.6% for CAVI as well as a previous report [20].

### Trunk flexibility as an index test and handgrip strength

On the day that arterial stiffness was determined, trunk flexibility was also assessed by a sit-and-reach test after stretching using a T-283 device (Toei Light, Tokyo, Japan), as described [5]. Independent experts in exercise testing who were blinded to other clinical information collected the data. The distance moved by the device was recorded. Participants sat on the floor, with their hips, back, and occipital region of the head touching a wall, with the legs held straight at 90° in front of the upper body. The zero point of the device was set in this position. They then bent forward slowly and reached as far forwards as possible. Two or more trials were performed, and then the average of the two highest values was taken as the definitive value.

Thereafter, handgrip strength was measured using a dynamometer (T.K.K.5001 Grip-A, Takei, Tokyo, Japan) with a precision of 0.1 kg [22]. The width of the handle was adjusted to fit each hand and the second phalanx rested against the inner stirrup to measure maximal strength three times. The elbow was fully extended because handgrip strength is influenced by elbow position. According to previous studies [23], [24], the highest values in the stronger hand were reported. We expressed handgrip strength data normalized to the body weight as follows: Handgrip strength (N/kg) = measured values×9.8/body weight. The constant 9.8 is the conversion factor from kg to N. In general, handgrip strength discriminates function in all adult age groups, predicts incident disability, and closely correlates with power in other muscle groups [25], [26]. The day-to-day CVs were 6.4±1.6% and 4.1±0.8% for the sit-and-reach test and handgrip strength, respectively, as determined in 10 individuals on two separate days.

### Physical activity

Habitual physical activity was assessed using the four-part International Physical Activity Questionnaire (IPAQ short form) translated into Japanese [27], [28]. The participants answered questions in the survey that included the number of days per week and minutes per day spent on 1) vigorous activity, 2) moderate activity, 3) walking for at least 10 min at a time, and 4) duration of sitting and/or lying down (excluding sleeping) per day. Scores for vigorous, moderate, and walking activity, as well as the duration of sitting, were calculated in hours per week. The hours per week for vigorous activity, moderate activity, or walking were multiplied by the average metabolic cost (METs) of 8, 4, or 2.5, respectively as described [27]. The sum of the daily energy expenditure due to participation in vigorous and moderate activity as well as walking was calculated as the total energy expenditure of the amount of physical activity (PA) (METs·hour/week). The high reliability and validity of this questionnaire have been established [28]. Several questions were not answered in the present study and some participants decided not to answer the questionnaire at all (n = 98). Thus, we could not collect physical activity data from all participants.

### Data analysis and statistics

Because we excluded all data with missing values from statistical analyses, data for the remaining 1,150 (Analyses-A and -C) or 1,052 (Analysis-B) participants were analyzed. These participants were assigned to groups based on age and sex as young, middle-aged, and older (ages 18 to 39, 40 to 59, and 60 to 84 y for men; ages 18 to 39, 40 to 59, and 60 to 89 y for women). The participants in each category were then assigned to groups with either poor- or high-flexibility based on the median values of the sit-and-reach test for every 10-year of age bracket from the teens to the eighties.

#### Analysis-A

Continuous data were analyzed by two-way ANOVA (category×flexibility) and ANCOVA that included height, weight, BMI, HR, MBP, smoking status, anti-hypertensive medication, anti-hyperlipidemic medication, handgrip strength, and surveyed area (Osaka, 1; Kumamoto, 2; Tokyo, 3) as a covariate. Significant F values were assessed using a post hoc test with the Bonferroni correction to identify significant differences among mean values. Differences in non-parametric variables were analyzed by the Kruskal-Wallis test and the Scheffé method. Relationships between trunk flexibility and arterial stiffness were assessed using Pearson's correlation.

#### Analysis-B

The influence of flexibility on arterial stiffness in all participants (n = 1,052), men (n = 488), women (n = 564), premenopausal (n = 276), and postmenopausal (n = 288) women was assessed by univariate regression analyses and stepwise multiple regression analyses. Variables for the stepwise linear regression model were selected based on univariate correlation analyses and variables that are known or thought to be associated with arterial stiffness from published observations such as sex (male, 1; female, 2) [5], [29]–[32]. Considering the collinearity of arterial stiffness and SBP, we did not include SBP and DBP [29], [31]. Moreover, because height might be influenced by age and sex, we did not include height and weight in multiple regression analysis of all participants.

#### Analysis-C

Recent studies have established CAVI≥8.0 as an optimal cut-off point for predicting carotid arteriosclerosis [18], [33]. We therefore assessed the performance of flexibility as a diagnostic test by calculating the sensitivity, specificity, and positive and negative predictive values of the sit-and-reach test at different cut-off values from receiver operating characteristic (ROC) curves to detect arterial stiffening using CAVI≥8.0. Optimal cut-off values of flexibility were defined as values that maximized sensitivity and specificity and thus minimized the expression of (1 - Sensitivity)^2^+(1 - Specificity)^2^. The CAVI calculated using the multiple regression equation obtained from multiple regression analysis was also assessed in all participants using ROC analysis. All data were statistically analyzed using SPSS for Windows 14.0J (IBM). Data are presented as means ± SEM. Differences were considered significant at P<0.05.

## Results

Table 1 shows the characteristics of the participants. Age, height, weight, BMI, HR, BP, and PP did not differ between the subgroups with high- and poor-flexibility (ANOVA indicated significant differences in height between both subgroups of middle-aged men and young women, but ANCOVA found no significant differences in height within all groups). Handgrip strength was higher in older men and older women with high flexibility. The amount of PA also did not differ between high- and poor-flexibility in all categories.

**Table 1:** Characteristics of the participants according to age, sex, and flexibility.

	Young		Middle-aged		Older	
	High	Poor	High	Poor	High	Poor
Men	n = 105	n = 105	n = 91	n = 92	n = 71	n = 72
Age, y	24±1	24±1	49±1*	49±1*	68±1* †	68±1* †
Height, cm	171.6±0.6	171.4±0.6	172.0±5.8	169.8±0.6‡	165.7±0.8* †	164.8±0.9* †
Weight, kg	67.2±1.0	65.6±1.0	72.8±1.1*	70.4±1.0*	65.1±1.0†	64.2±1.0†
BMI, kg/m^2^	22.8±0.3	22.3±0.3	24.6±0.4*	24.4±0.3*	23.7±0.3*	23.6±0.3*
HR, beats/min	64±1	64±1	65±1	65±1	66±1*	69±2*
SBP, mmHg	126±1	125±1	134±2*	130±2*	137±2* †	137±2* †
DBP, mmHg	73±1	73±1	86±1*	84±1*	84±1*	84±1*
MBP, mmHg	91±1	91±1	102±1*	99±1*	102±1*	102±1*
PP, mmHg	53±1	52±1	47±1*	46±1*	53±2†	53±2†
Current smoker, n/%	4/3.8	13/12.3	13/14.2	7/7.6	4/5.6	5/6.9
A-hypertensive, n/%	0/0.0	0/0.0	7/7.7	6/6.5	15/21.1*	25/34.7* †
A-hyperlipidemic, n/%	0/0.0	0/0.0	1/1.1	4/4.3	3/4.2	5/6.9
Handgrip, N/kg	6.7±0.1	6.6±0.1	6.0±0.1*	6.0±0.1*	6.1±0.1*	5.7±0.1* ‡
	n = 95	n = 94	n = 74	n = 87	n = 68	n = 70
PA, MET·h/week	46.5±3.8	37.3±3.4	28.6±3.2*	27.4±3.1*	37.8±4.0	33.1±3.8
Women	n = 99	n = 101	n = 108	n = 110	n = 98	n = 98
Age, y	24±1	25±1	50±1*	50±1*	69±1* †	70±1* †
Postmenopausal, n/%	0/0.0	0/0.0	55/50.9	57/51.8	98/100.0	98/100.0
Height, cm	159.9±0.5	158.1±0.5‡	158.3±0.5	158.0±0.5	152.4±0.5* †	151.6±0.6* †
Weight, kg	54.0±0.8	51.7±0.7	52.4±0.8	53.5±0.8	51.6±0.7	51.9±0.7
BMI, kg/m^2^	21.1±0.3	20.7±0.2	20.9±0.3	21.4±0.3	22.2±0.3* †	22.6±0.3* †
HR, beats/min	65±1	68±1	65±1	66±1	69±1†	71±1†
SBP, mmHg	115±1	114±1	123±2*	124±1*	134±2* †	135±2* †
DBP, mmHg	69±1	69±1	79±1*	78±1*	80±1*	81±1*
MBP, mmHg	84±1	84±1	93±1*	93±1*	98±1* †	99±1* †
PP, mmHg	46±1	45±1	44±1	46±1	53±1* †	54±1* †
Current smoker, n/%	1/1.0	3/3.0	8/7.4	8/7.3	0/0.0	3/3.1
A-hypertensive, n/%	0/0.0	0/0.0	2/1.9	5/4.5	25/25.5* †	26/26.5* †
A-hyperlipidemic, n/%	0/0.0	0/0.0	0/0.0	6/5.5	13/13.3* †	14/14.3*
Handgrip, N/kg	5.1±0.1	5.1±0.1	5.3±0.1	5.2±0.1	4.8±0.1* †	4.4±0.1* † ‡
	n = 89	n = 88	n = 98	n = 102	n = 93	n = 94
PA, MET·h/week	26.3±3.1	31.5±3.4	39.8±4.3	30.4±3.4	39.1±3.8	36.8±3.8

Data are means ± SE. BMI, body mass index; HR, Heart rate; SBP, Systolic blood pressure; DBP, diastolic pressure; MBP, mean blood pressure; PP, pulse pressure; A-hypertensive, Anti-hypertensive medication; A-hyperlipidemic, Anti-hyperlipidemic medication; Handgrip, Handgrip strength; PA, amount of physical activity.

*P<0.05 vs. young;

† P<0.05 vs. middle-aged;

‡ P<0.05 vs. high-flexibility within the same age and sex category.

ANOVA showed a significant difference in height between groups of middle-aged men and young women with high- and poor-flexibility, but ANCOVA did not.

**Figure 2:**
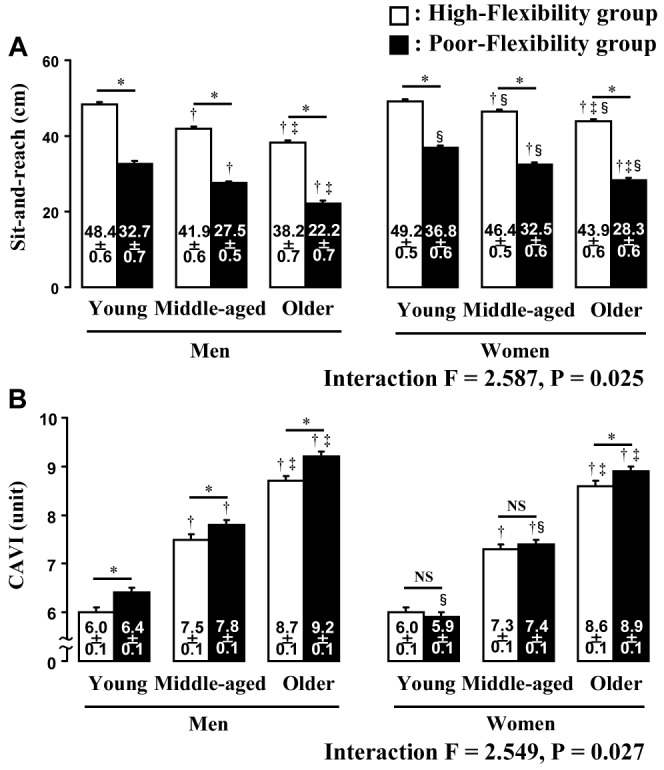
Trunk flexibility (A) and arterial stiffness (B) in groups with high- or poor-flexibility. CAVI, cardio-ankle vascular index; ^*^P<0.05 vs. high-flexibility within the same age category; ^†^P<0.05 vs. young within the same sex and flexibility group; ^‡^P<0.05 vs. middle-age within the same sex and flexibility group; ^§^P<0.05 vs. men within the same flexibility and age category; NS, not statistically significant.

Figure 2 shows trunk flexibility (A) and arterial stiffness (B) in groups with high- and poor-flexibility within each age and sex category. Sit-and-reach values were lower in the groups in each age and sex category with poor-flexibility, in middle-aged and older, than in younger participants and in older, than in middle-aged participants. On the other hand, the CAVI was higher in middle-aged and older, than in younger participants within both flexibility groups and also was notably higher in older, than in middle-aged participants. Most importantly, in all age categories of men from young to older, the CAVI was higher in poor-flexibility, than in high-flexibility, but did not differ between women with high- and poor-flexibility in any age category except for older women. In addition, CAVI was lower in young and middle-aged women with poor-flexibility than in coeval men with poor-flexibility. The results remained significant after normalizing CAVI for height, weight, BMI, HR, MBP, smoking status, anti-hypertensive medication, anti-hyperlipidemic medication, handgrip strength, and survey area when analyzed by ANCOVA.

**Figure 3:**
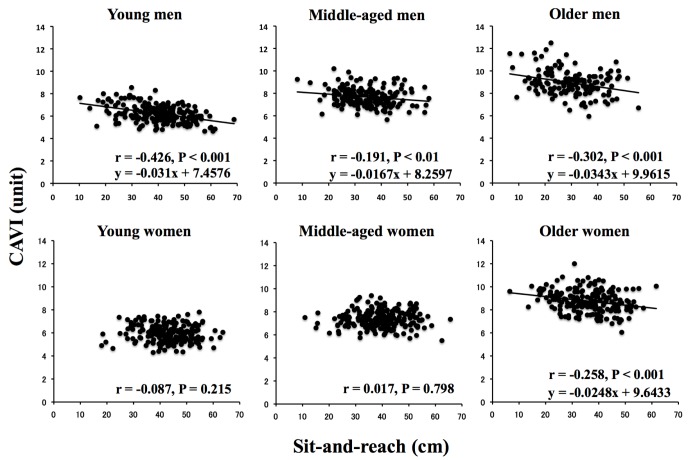
Relationships between trunk flexibility and arterial stiffness (CAVI) according to age and sex. CAVI, cardio-ankle vascular index, which is theoretically adjusted by BP.

Figure 3 shows the relationships between sit-and-reach and CAVI in each age and sex category. The CAVI significantly correlated with sit-and-reach in all men and older women categories. However, correlations were not significant in young and middle-aged women.

The findings of the univariate regression analysis indicated that sit-and-reach scores correlated with age, BMI, MBP, HR, handgrip strength, amount of PA. We also found that the CAVI correlated with age, height, BMI, MBP, HR, handgrip strength. We thus performed a stepwise multiple-regression analysis of the data from all participants. Age, sex, sit-and-reach, BMI, and MBP were the only independent factors modulating CAVI (Table 2). However, HR, handgrip strength, amount of PA, surveyed area, anti-hypertensive medication, anti-hyperlipidemic medication, and smoking status did not significantly affect the multiple regression analysis. In addition, sit-and-reach was a second independent factor modulating CAVI in men, but a fourth independent factor in women, indicating that trunk flexibility contributes less to arterial stiffness in women (β = −0.044) than in men (β = −0.142). Importantly, multiple regression analysis selected flexibility as a significant independent factor modulating CAVI in postmenopausal women and in men, but not in premenopausal women.

**Table 2:** Stepwise multiple-regression analyses of factors affecting arterial stiffness.

	Regression Coefficient	SE	β	P	R^2^ Change (%)
All participants (n = 1052)^1^					
Constant	5.977	0.267		<0.001	
Age	0.060	0.001	0.824	<0.001	72.3
Sex	−0.300	0.049	−0.106	<0.001	1.1
Flexibility	−0.012	0.002	−0.086	<0.001	0.6
BMI	−0.040	0.008	−0.090	<0.001	0.6
MBP	0.005	0.002	0.043	<0.020	0.1
Men (n = 488)^2^					
Constant	3.959	0.979		<0.001	
Age	0.058	0.002	0.786	<0.001	71.4
Flexibility	−0.019	0.004	−0.142	<0.001	1.5
BMI	−0.042	0.011	−0.089	<0.001	0.6
MBP	0.009	0.003	0.073	0.006	0.4
Height	0.010	0.005	0.050	0.049	0.2
Women (n = 564)^3^					
Constant	5.179	0.270		<0.001	
Age	0.065	0.002	0.897	<0.001	75.2
BMI	−0.085	0.021	−0.185	0.001	0.5
Weight	0.022	0.008	0.122	0.005	0.3
Flexibility	−0.006	0.003	−0.044	0.045	0.2
Premenopausal women (n = 276)^4^					
Constant	5.983	0.361		<0.001	
Age	0.060	0.003	0.741	<0.001	56.6
HR	−0.012	0.003	−0.138	<0.001	2.0
BMI	−0.032	0.012	−0.102	0.008	1.0
Postmenopausal women (n = 288)^5^					
Constant	−3.797	1.544		0.015	
Age	0.092	0.006	0.805	<0.001	47.6
Height	0.049	0.009	0.286	<0.001	3.6
Flexibility	−0.013	0.005	−0.120	0.005	1.1
Weight	−0.016	0.006	−0.118	0.008	1.2

1 Excluded variables: HR, Handgrip strength, Amount of PA, Surveyed area, A-hypertensive-m, A-hyperlipidemic-m, and Smoking (R^2^ = 0.747, Adjusted R^2^ = 0.745, P<0.001).

2 Excluded variables: Weight, HR, Handgrip strength, Amount of PA, Surveyed area, A-hypertensive-m, A-hyperlipidemic-m, and Smoking (R^2^ = 0.741, Adjusted R^2^ = 0.738, P<0.001).

3 Excluded variables: Height, MBP, HR, Handgrip strength, Amount of PA, Surveyed area, A-hypertensive-m, A-hyperlipidemic-m, and Smoking (R^2^ = 0.761, Adjusted R^2^ = 0.760, P<0.001).

4 Excluded variables: Height, Weight, MBP, Flexibility, Handgrip strength, Amount of PA, Surveyed area, A-hypertensive-m, A-hyperlipidemic-m, and Smoking (R^2^ = 0.596, Adjusted R^2^ = 0.591, P<0.001).

5 Excluded variables: BMI, MBP, HR, Handgrip strength, Amount of PA, Surveyed area, A-hypertensive-m, A-hyperlipidemic-m, and Smoking (R^2^ = 0.535, Adjusted R^2^ = 0.529, P<0.001).

Sex: male, 1; female, 2. A-hypertensive-m, Anti-hypertensive medication; A-hyperlipidemic-m, Anti-hyperlipidemic medication.

Table 3 shows the performance of flexibility as a diagnostic test. The findings in men and women were significant. The sensitivity and specificity were >60%. We also conducted ROC curve analysis of the CAVI value estimated from multiple regression equations. The results were significant, with sensitivity and specificity >80%.

**Table 3:** Application of flexibility as a diagnostic test.

	n	Cut-off point	AUC (95%CI)	Se (%)	Sp (%)	PPV (%)	NPV (%)
ROC curve analyses of flexibility							
All participants	1150	39.0 cm	0.674 (0.642–0.707)	70.5	57.0	46.3	78.6
Men	536	33.2 cm	0.711 (0.666–0.756)	61.7	69.7	53.4	76.4
Women	614	39.2 cm	0.639 (0.592–0.686)	61.1	62.0	44.3	76.3
ROC curve analysis of CAVI estimated from multiple regression equation for all participants							
All participants	1150	7.9 unit	0.930 (0.916–0.944)	85.9	84.9	74.9	92.0

AUC, area under the curve; CI, confidence interval; Se, sensitivity; Sp; specificity; PPV, positive predictive value; NPV, negative predictive value; ROC curve, receiver operating characteristic curve; CAVI, cardio-ankle vascular index.

## Discussion

The salient findings are as follows. Compared with the high-flexibility groups, arterial stiffness by CAVI was greater in the poor-flexibility groups of all age categories of men and older women. The CAVI significantly correlated with trunk flexibility in all categories of men and in older women. However, significant differences and correlations were not found among young and middle-aged women. The findings of the ROC curve analysis indicated that the results for men and women were significant and that the sensitivity and specificity were both >60%. To our knowledge, this is the first study to evaluate sex differences in flexibility-arterial stiffness relationship after adjusting for major confounding factors and to develop a simple method of assessing arterial stiffness by means of flexibility.

Previous studies have established that BP affects PWV as an index of arterial stiffness [6]. Here, we assessed arterial stiffness using the CAVI, which is theoretically adjusted by BP [20], and no significant differences in BP were found between groups with high- and poor-flexibility in each age and sex category. We also normalized arterial stiffness for BP using ANCOVA and multiple regression analysis. Nevertheless, the results indicated that all men and older women with poor-flexibility had stiffer arteries than those with high-flexibility and that sit-and-reach scores significantly correlated with the CAVI. Therefore, trunk flexibility appears to be a significant predictor of arterial stiffness after adjustment for BP, which is a major confounding factor.

Contrary to the present results, one previous study found that flexibility is not significantly related to arterial stiffness in young persons [5]. However, they did not analyze this relationship in each sex [5], which could explain the conflicting findings between their findings and those of the present study. We assessed relationships between flexibility and arterial stiffness among age and sex categories and found that flexibility was significantly related to arterial stiffness in all men from young to older and in older women, but not in young and in middle-aged women. Yamamoto et al. also performed a stepwise-regression analysis among age and components of fitness, so that sit-and-reach is significantly independently correlated with arterial stiffness. Their findings indicate that the statistical contribution of sit-and-reach (β = −0.14) to arterial stiffness compares favorably with that of peak oxygen uptake (β = −0.12) [5]. Previous studies indicate that regular stretch exercise might reduce arterial stiffness without increasing maximum oxygen uptake (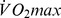) [4] and that arterial stiffness in young and middle-aged men does not significantly differ among participants of different 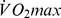 levels [34]. Thus, it seems unlikely that arterial stiffness is simply modulated by 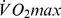 than flexibility. Similarly, in this study, ANCOVA uncovered no significant differences in age, height, weight, BMI, HR, PP, and BP between groups with high- and poor-flexibility in each age and sex category. In particular, within the young and middle-aged categories, no significant differences were observed in handgrip strength, which closely correlates with power in other muscle groups [25], [26]. Because we used a self-reported measure of physical activity, which the hours for physical activity were multiplied by the average METs, it may be hard to compare amount of PA among age and sex categories. However, in each age and sex category, amount of PA did not differ between groups with high- and poor-flexibility. Thus, our data of physical characteristics, handgrip strength, and amount of PA mean that such parameters of participants without sit-and-reach score and arterial stiffness are similar between groups with high- and poor-flexibility, particularly within the young and middle-aged categories. Furthermore, multiple regression analysis of all participants revealed both flexibility and sex as significant independent factors modulating arterial stiffness, although handgrip strength and the amount of PA did not significantly enter the equation. In contrast to the findings for men and postmenopausal women, multiple regression analysis did not select flexibility as a significant independent factor modulating arterial stiffness in premenopausal women. Therefore, our results indicated that sex differences exist in flexibility-arterial stiffness relationship.

Previous study has demonstrated that the CAVI of healthy men and women without cardiovascular risk factors increases almost linearly with age from 20 to 70 years [17]. We applied the linear regression equation to the average CAVI values, and then estimated “vascular age” for each group. Vascular age differed from 6 to 9 years between all adult men and older women with high- and poor-flexibility. Therefore, these findings imply that the differences are not only statistically significant, but also clinically meaningful.

The physiological mechanisms underlying the relationship between flexibility and arterial stiffness have not yet been elucidated. Our data indicated that flexibility is a significant predictor of arterial stiffness after adjusting for the influences of BP and thus vascular structural and/or functional factors may mainly contribute to the relationship. One possibility is that both arterial stiffness and flexibility are structurally determined by a specific composition of muscles or connective tissues such as the elastin and collagen [3]. Thus, age-related changes in arterial stiffness might correspond to age-related alterations in flexibility within the same individual as previously considered [5]. Another possibility is that arterial stiffness is functionally determined by arterial vascular tone, and that tone is partially regulated by sympathetic nerve activity [3]; if so, then the same neural factors might contribute to the regulation of flexibility as well as arterial stiffness. However, direct evidence derived from studies *in vivo* is not available to support this contention.

We can only speculate about the mechanisms responsible for sex differences in the relationship between trunk flexibility and arterial stiffness. In general, arterial stiffness is significantly lower in premenopausal women than in age-matched men, whereas this difference disappears when women reach menopause [8], [9]. Because estrogen has potent vasodilatory and antiarteriosclerosis effects in vascular tissue [35], [36], an estrogen deficiency in postmenopausal women brings about a rapid increase in arterial stiffness [8], [9]. Taken together with these findings, our results suggest that the powerful ability of estrogen to reduce arterial stiffness might cancel out the relationship in premenopausal women, and thus the difference and the correlation might be observed only in postmenopausal women. Further studies of assessing estrogen will be needed to verify these notions.

We further examined the performance of flexibility as a diagnostic test to predict the presence of arterial stiffening (CAVI≥8.0) [18], [33]. The sit-and-reach test is simple to administer, convenient to measure, and does not require specific skills or expert knowledge compared with other methods of assessing arterial stiffness. Here, we analyzed only healthy participants without overt chronic diseases, and mean or median values of flexibility at each age and sex category approximately corresponded to average physical fitness score levels in the most recent annual physical fitness test published by the Japanese Ministry of Education, Science, and Culture. Therefore, although the accuracy of assessment is not always extremely high and further controlled clinical and diagnostic accuracy studies are needed, the present results imply that testing the trunk flexibility of individuals without overt chronic diseases could simplify predictions of arterial stiffening because it can be implemented anywhere.

The present study used the sit-and-reach test as an indicator of flexibility as described [5]. This test might be differentially influenced by arm and leg length, but the individual zero point for each participant minimized the influences of arm and leg length. The abdominal obesity also limits ability to perform the sit-and-reach test. However, because we excluded participants with BMI>30 kg/m^2^ and then adjusted for BMI, our analyzed data did not include participants with the largest waist. Indeed, no significant differences in weight and BMI were found between high- and poor-flexibility in each age and sex category. The sit-and-reach test has been commonly used to assess flexibility from the viewpoint of health-related fitness. Thus, our data of sit-and-reach test are actually considered to reflect trunk flexibility.

**Figure 4:**
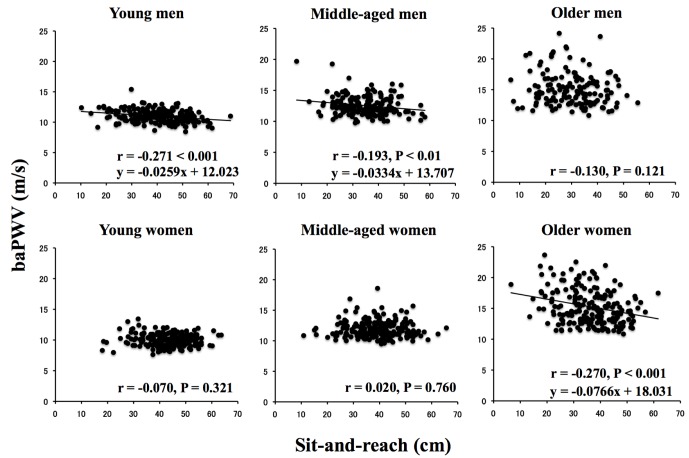
Relationships between trunk flexibility and arterial stiffness (baPWV) according to age and sex. baPWV, brachial-ankle PWV as an index of stiffness in the central arteries.

In this study, we firstly aimed to examine the involvement of BP in flexibility-arterial stiffness relationship and thus assessed arterial stiffness using the CAVI [20]. Previous reports have clearly demonstrated that CAVI is significantly associated with many arteriosclerotic diseases and the risk for cardiovascular diseases [15], [17], [21], [33]. However, the CAVI provides information about overall arterial stiffness, including the aorta, femoral artery, and tibial artery, and thus does not provide information about only central arterial stiffness. Hence, to employ more gold-standard method of quantifying arterial stiffness, we also calculated brachial-ankle PWV (baPWV), which mainly reflects stiffness in the central arteries [37]–[39], and these data showed a similar result with the CAVI, namely sex differences exist in flexibility-arterial stiffness relationship (Fig. 4). In addition, because vascular length was automatically calculated according to the participant' height, the CAVI may be affected by height as well as by baPWV [30]. We thus removed the influence of height on the results using ANCOVA and multiple regression analysis [32]. Therefore, although we could not employ the gold-standard method such as carotid-femoral PWV, our CAVI data are believed to provide information about qualitatively similar to baPWV, which mainly reflects central arterial stiffness. Further studies also will be needed to compare the data between CAVI and carotid-femoral PWV if the high availability of CAVI is shown.

Several important limitations require emphasis. First, 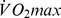, leg power, central adiposity (*i.e.*, waist circumference), and blood data were not determined although these factors are known to affect arterial stiffness or flexibility. Therefore, we could not completely discuss the involvement of such factors in flexibility-arterial stiffness relationship. In particular, because we did not measure circulating estrogen concentrations in addition to risk factors for arteriosclerosis such as blood lipids, the effects of estrogen on vascular tissue and the physiological mechanisms of sex differences in flexibility-arterial stiffness relationship are unclear. Second, we did not monitor menstrual phases among the premenopausal participants. Arterial stiffness fluctuates with the phases of the menstrual cycle [40]. Thus, whether a relationship between trunk flexibility and arterial stiffness could be identified even in young and middle-aged women if all premenopausal women in this population were tested during the early follicular phase remains unclear. Third, to keep our results as simple as possible, we used two-way ANOVA and ANCOVA. Although three-way ANOVA and ANCOVA (age×sex×flexibility) indicated a somewhat significant interaction, this analysis has a high likelihood of more complicated data interpretation and we did not use such methods. Finally, the cross-sectional study design limited our ability to infer a cause-and-effect relationship between trunk flexibility and arterial stiffness.

In summary, our results indicate that flexibility-arterial stiffness relationship is not affected by BP, which is a major confounding factor. In addition, sex differences are observed in flexibility-arterial stiffness relationship. That is, poor trunk flexibility increases arterial stiffness in young, middle-aged, and older men, whereas the relationship in women is found only in the elderly. Also, the trunk flexibility test can help to assess arterial stiffening at home or elsewhere. The appropriate sit-and-reach cut-off values for men and women were 33.2 and 39.2 cm, respectively.
